# Fermented *Houttuynia cordata* Juice Exerts Cardioprotective Effects by Alleviating Cardiac Inflammation and Apoptosis in Rats with Lipopolysaccharide-Induced Sepsis

**DOI:** 10.3390/nu17030501

**Published:** 2025-01-29

**Authors:** Natticha Sumneang, Anongporn Kobroob, Sukanya Phungphong, Worakan Boonhoh, Chuchard Punsawad, Napapan Kangwan

**Affiliations:** 1 Department of Medical Science, School of Medicine, Walailak University, Nakhon Si Thammarat 80160, Thailand; natticha.su@wu.ac.th (N.S.); sukanya.pu@wu.ac.th (S.P.); chuchard.pu@wu.ac.th (C.P.); 2 Research Center in Tropical Pathobiology, Walailak University, Nakhon Si Thammarat 80160, Thailand; 3 Division of Physiology, School of Medical Sciences, University of Phayao, Phayao 56000, Thailand; anongporn.ko@up.ac.th; 4 Akkhraratchakumari Veterinary College, Walailak University, Nakhon Si Thammarat 80160, Thailand; worakan.bo@wu.ac.th

**Keywords:** fermented *Houttuynia cordata* juice, heart, anti-inflammation, antioxidant, apoptosis

## Abstract

Background/Objectives: Sepsis-induced cardiac dysfunction is a major problem that often leads to severe complications and a poor prognosis. Despite the growing awareness of its impact, effective treatment options for sepsis-induced cardiac dysfunction remain limited. To date, fermented products of *Houttuynia cordata* (HC), known for its rich bioactive properties, have shown potential in modulating inflammatory and oxidative stress pathways. However, treatment with fermented HC juice (FHJ) in lipopolysaccharide (LPS)-induced sepsis in rats has not been investigated. Methods: Rats were pretreated with FHJ at doses of 200 mg/kg and 400 mg/kg for 2 weeks. After that, the rats were injected with a single dose of LPS (10 mg/kg), and 12 h after injection, they developed sepsis-induced cardiac dysfunction. Then, cardiac function, oxidative stress, inflammation, apoptosis, and cardiac injury markers were determined. Results: Pretreatment with FHJ at doses of 200 mg/kg and 400 mg/kg prevented LPS-induced cardiac dysfunction in rats by attenuating cardiac inflammation (IL-1β, TLR-4, and NF-κB levels), oxidative stress (MDA levels), and apoptosis (cleaved-caspase 3 and Bax/Bcl-2 expression) and reducing markers of cardiac injury (LDH and CK-MB levels). Conclusions: These results suggest that FHJ could be a potential therapeutic agent for sepsis-induced heart disease.

## 1. Introduction

Sepsis, a systemic inflammatory response, has become a global concern, with an estimated prevalence of 48.9 million cases and 11 million sepsis-related deaths worldwide in 2017, accounting for almost 20% of all deaths worldwide [1,2]. There are numerous factors in our daily lives that lead to susceptibility to sepsis, such as diabetes, trauma, prolonged hospitalization, and aging [3]. Nonetheless, cardiovascular disease is a consequence of sepsis and is an emerging health problem [4,5,6]. Evidence shows that approximately 50% of septic patients had deleterious cardiac performance, characterized by impaired cardiac contractility and diastolic function, as indicated by the percentage of left ventricular (LV) ejection fraction and fractional shortening (%LVEF and %LVFS, respectively) [6,7,8,9,10].

Since cardiac dysfunction is a complication that occurs in septic patients with excessive inflammatory response, it was observed in a rodent model with lipopolysaccharide (LPS)-induced sepsis [11,12,13,14]. LPS, a component of Gram-negative bacteria, plays an important role in the pathophysiology of the inflammatory response through Toll-like receptor 4 (TLR-4), one of the strongest receptors of the innate immune system [15,16,17,18]. Upon binding of LPS to TLR-4, the nuclear factor kappa-light-chain enhancer of activated B cells (NF-κB) signaling pathway is initiated to trigger intracellular adaptor molecules and release inflammatory cytokines (e.g., tumor nuclear factor alpha (TNF-α), interleukin 1 beta (IL-1β), IL-6, etc.) [19,20]. This excessive recruitment of inflammatory cytokines into the system and heart during cardiac injury has been reported to result in vulnerability to myocardial damage and adverse cardiac events [21].

In addition to inflammation, oxidative stress is also one of the pathological hallmarks associated with LPS-induced cardiac damage [22,23,24]. LPS has been reported to disrupt the balance between free radicals and antioxidants, leading to an overwhelming production of reactive oxygen species (ROSs) and, thus, to oxidative stress in various organs of sepsis models, especially the heart [23,25,26,27]. In addition, the cardiac cell death modality, apoptosis, has been reported to be observed in LPS-induced sepsis in rodent models [22,23,28]. Apoptosis is commonly considered a programmed cell death process in response to organ injury [29]. The mechanism of apoptosis is mainly triggered by two pathways: the intrinsic and the extrinsic pathways [30]. The intrinsic pathway is associated with molecule-mediated mitochondrial damage such as oxidative stress, while the extrinsic pathway refers to the binding of molecular ligands to death receptors such as TNF-α and TNF receptors [30,31]. Numerous studies have shown that cardiac apoptosis induced by sepsis contributes to pathological changes in the myocardium and impaired myocardial function [28,32,33]. Therefore, inflammation or oxidative stress is suggested to result in an influence on cardiac apoptosis induced by LPS-induced sepsis in rodent models. Direct modulation of inflammation and oxidative stress is considered a target for therapeutic exploration to further suppress the apoptosis modality in sepsis-induced cardiac dysfunction.

*Houttuynia cordata* Thunb., known in Thailand as Plukaow, is a herbaceous perennial plant native to Southeast Asia [34,35]. To date, there are commercially available products for human consumption, such as fermented products or tablets, as dietary supplements [34,35]. The fermentation process remarkably enhances the therapeutic properties of *Houttuynia cordata* (HC) by increasing the concentration and bioavailability of its bioactive compounds [36]. The fermented products of HC show promising potential in various applications, particularly in the treatment of inflammatory diseases, in the modulation of the immune system, and as cosmetic products [35,37,38]. As they contain several chemical constituents, such as alkaloids, essential oils, phenolic acids, and flavonoids, they are used to treat various diseases, and they include antibacterial, antiviral, anti-allergic, anticancer, and antidiabetic activities [34,35,39,40,41,42,43,44,45]. Although previous studies have demonstrated that fermented products of HC have the ability to potently suppress both inflammation and oxidative stress in diabetic rats and LPS-induced macrophage cells, models that are potentially involved in susceptibility to sepsis [34,35], treatment with products of fermented HC juice (FHJ) has been not investigated in terms of the direct effects of sepsis-induced myocardial damage, including effects on cardiac function, cardiac inflammation, oxidative stress, and cardiac apoptosis, in a rat model. Therefore, this study aimed to investigate the effects of FHJ on cardiac inflammation, oxidative stress, cardiac apoptosis, and cardiac function in LPS-induced cardiac impairment in rats.

## 2. Materials and Methods

### 2.1. Preparation of Fermented Houttuynia cordata Thunb. Juice

FHJ was obtained from Rincome Group Company Limited, Sansai District, Chiang Mai Province, Thailand (product lot number: 21/03/2022) [34]. Five hundred milliliters of FHJ was filtered through Whatman No. 1 filter paper and lyophilized to obtain the FHJ. The FHJ was protected from light and stored at −20 °C for further investigation. The FHJ was lyophilized to obtain a powder that was then reconstituted with distilled water to produce concentrations of 200 mg/kg and 400 mg/kg for experimental use.

### 2.2. Animal Preparation

Thirty-two male Wistar rats weighing 200–220 g were purchased from Nomura Siam International Co., Ltd. (Bangkok, Thailand). All rats were accommodated in a controlled environment, maintaining a 12 h dark/light cycle and a temperature range of 22 °C to 24 °C. The rats had ad libitum access to a standard chow diet and water. After one week of acclimatization, the rats were randomly assigned to four groups (*n* = 8/each group), including (1) the NSS group (vehicle received in a normal saline solution, i.p.), (2) the LPS group (10 mg/kg in NSS, i.p.), (3) FHJ (200 mg/kg) + LPS (10 mg/kg in NSS, i.p.; FHJ200-LPS group), and (4) FHJ (400 mg/kg) + LPS (10 mg/kg in NSS, i.p.; FHJ400-LPS group). The rats received their designated pretreatment with FHJ via daily oral gavage feeding for 14 consecutive days before LPS injection. The doses of 200 mg/kg and 400 mg/kg of FHJ were selected based on our preliminary data using a modified dose from Sakuludomkan et al. [34]. Regarding the protocol for the LPS groups, previous studies demonstrated that rats injected with a single dose of 10 mg/kg of LPS exhibited impaired cardiac function at 12 h post-injection [11,46].

**Figure 1:**
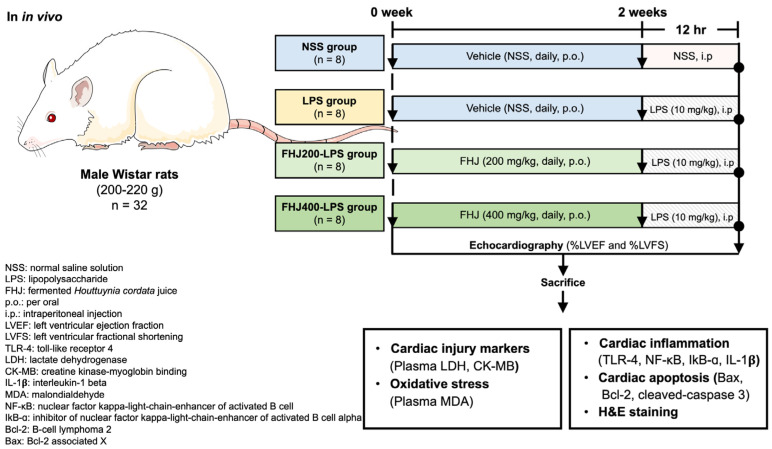
Schematic overview of the experimental protocol. NSS: normal saline solution; FHJ: fermented *Houttuynia cordata* juice; p.o.: per oral; i.p.: intraperitoneal injection; LVEF: left ventricular ejection fraction; LVFS: left ventricular fractional shortening; TLR-4: toll-like receptor 4; LDH: lactate dehydrogenase; CK-MB: creatine kinase–myoglobin binding; IL-1β: interleukin-1 beta; MDA: malondialdehyde; NF-κB: nuclear factor kappa-light-chain-enhancer of activated B cells; IκB-α: inhibitor of nuclear factor of kappa-light-chain-enhancer in B-cell alpha; Bcl-2: B-cell lymphoma 2; Bax: Bcl-2-associated X.

At the end of the study protocol, after a 12 h LPS injection, the rats were anesthetized using thiopental anesthesia (50 mg/kg, i.p.) to determine their cardiac function using echocardiography. Subsequently, the rats were kept anesthetized with thiopental and then sacrificed. Their blood was collected to determine cardiac injury markers and systemic oxidative stress. The heart was rapidly removed for molecular analysis, which included analyses of cardiac inflammation, cardiac apoptosis, and cardiac structural staining. The experimental protocol is illustrated in Figure 1.

### 2.3. Cardiac Performance Determination

Echocardiography (Mindray DC-70 Ultrasound System, Shenzhen, China) was used to analyze LV function under light anesthesia. The level of papillary muscle was identified, and M-mode echocardiographic images were recorded to determine the %LVEF and %LVFS. Each measurement involved using the average of three consecutive cardiac cycles, with the operator being blinded to the treatment assignment.

### 2.4. Plasma Lactate Dehydrogenase (LDH) Determination

Plasma LDH was used to indicate tissue damage [47]. LDH in the plasma was determined with an LDH toxicity test (Roche Cobas, Roche, Switzerland) using an automated biochemical system. The LDH activity was assessed according to the manufacturer’s instructions. Briefly, the plasma LDH catalyzed the conversion of L-lactate into pyruvate, resulting in NADH production. The rate of NADH formation is directly proportional to the catalytic LDH activity. Elevated absorbance is indicative of increased LDH activity in the plasma.

### 2.5. Plasma Creatine Kinase–Myoglobin Binding (CK-MB) Determination

Myocardial injury was indicated by the catalytic activity of CK-MB in plasma. It was measured within 24 h after blood collection using the isoenzyme CK-MB with an automated biochemical analyzer (Roche Cobas, Roche, Switzerland) according to the manufacturer’s instructions.

### 2.6. Plasma Malondialdehyde (MDA) Determination

Oxidative stress was determined using thiobarbituric acid reactive substances (TBARSs) based on the formation of MDA, which produces a pink color in plasma with thiobarbituric acid. Plasma TBARS levels were measured using the TBARS assay kit (Cayman Chemical, MI, USA) according to the manufacturer’s instructions. The concentration of plasma TBARSs was calculated from the standard curve, and the data are expressed as plasma MDA levels.

### 2.7. Hematoxylin and Eosin (H&E) Staining

The tissues of the LV were selected to assess histopathological changes, as they are representative of cardiac function. The LV tissues were placed in 4% formaldehyde solution for perfusion fixation. Then, the tissues were dehydrated and embedded in paraffin for sectioning slides and stained with H&E. Images of H&E staining were acquired under an inverted microscope (Eclipse Ts2, Nikon, Japan). Representative pictures of H&E-stained heart tissues were randomly selected in a field/sample-blind manner.

### 2.8. Western Blot Analysis

Protein lysates were prepared from cardiac tissues and loaded onto 10% sodium dodecyl sulfate–polyacrylamide gels for separation. Next, separated proteins were blotted onto a nitrocellulose membrane in a glycine/methanol-transfer buffer in a Wet/Tank blotting system (Bio-Rad, Hercules, CA, USA). The membranes were blocked for 1 h with either 5% bovine serum albumin (BSA) or 5% skimmed milk in Tris Buffer Saline with Tween 20 (TBST) and kept overnight at 4 °C with primary antibodies, as specified in Table 1. The membranes were then incubated with secondary antibodies for 1 h. The peroxidase reaction product was visualized using enhanced chemiluminescence detection reagents (Bio-Rad, USA). Western blotting was conducted using a ChemiPRO Imaging System XS (Cleaver Scientific Ltd., Warwickshire, UK), and the data were analyzed using the Image J program.

### 2.9. Statistical Analysis

Statistical analysis was performed using the GraphPad Prism 8 software (GraphPad Software, La Jolla, CA, USA). Data are expressed as the mean ± standard error of the mean (S.E.M). The normality of all data parameters was assessed using the Kolmogorov–Smirnov test. Differences among groups were analyzed using one-way ANOVA followed by the LSD post hoc test. A *p*-value less than 0.05 was considered statistically significant.

## 3. Results

### 3.1. FHJ Prevented LV Dysfunction in Rats with LPS-Induced Sepsis

**Figure 2:**
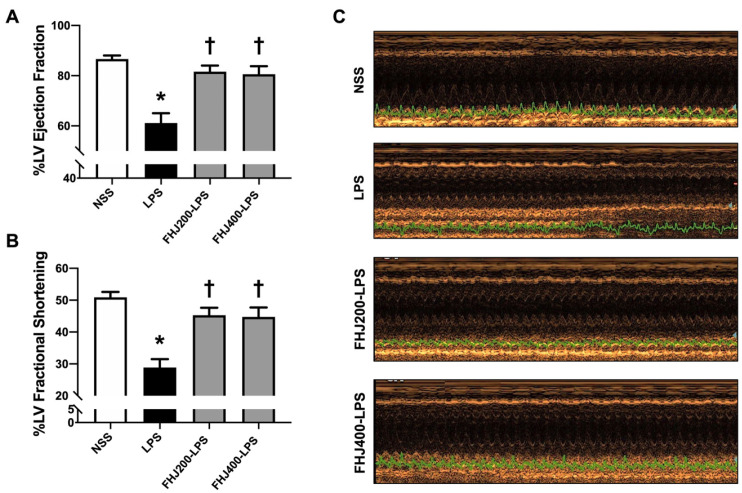
Effects of LPS injection and pretreatment with FHJ at 200 and 400 mg/kg for 2 weeks on LV function. (**A**) %LVEF, *n* = 8/group; (**B**) %LVFS, *n* = 8/group; (**C**) M-mode echocardiographic images in a parasternal short-axis view. * *p* < 0.05 vs. NSS and ^†^ *p* < 0.05 vs. LPS (one-way ANOVA followed by the LSD post hoc test). Data are expressed as means ± S.E.M. NSS: rats treated with vehicle; LPS: rats treated with a 12 h LPS injection; FHJ200-LPS: rats pretreated with 200 mg/kg of FHJ for 2 weeks followed by a 12 h LPS injection; FHJ400-LPS: rats pretreated with 400 mg/kg of FHJ for 2 weeks followed by a 12 h LPS injection. LVEF: left ventricular ejection fraction; LVFS: left ventricular fractional shortening; FHJ: fermented *Houttuynia cordata* juice.

We first assessed the LV function using echocardiography. After 12 h of administration of LPS via intraperitoneal injection, LV dysfunction was observed in these rats, as indicated by reductions in %LVEF and %LVFS in the LPS group compared with the normal rats in the NSS group (Figure 2A,B). This suggests that after 12 h, LPS injection successfully impaired cardiac function in rats. However, pretreatment with FHJ at doses of 200 mg/kg and 400 mg/kg significantly increased %LVEF and %LVFS back to normal levels after a 2-week pretreatment in LPS-injected rats (Figure 2A,B). This suggested that pretreatment with FHJ at 200 mg/kg and 400 mg/kg for 2 weeks resulted in a cardioprotective effect on LV function in rats with LPS-induced sepsis. Representative echocardiographic M-mode images of the NSS and LPS groups and those pretreated with FHJ at 200 mg/kg and 400 mg/kg are displayed in Figure 2C.

### 3.2. FHJ Reduced Cardiac Injury in Rats with LPS-Induced Sepsis

**Figure 3:**
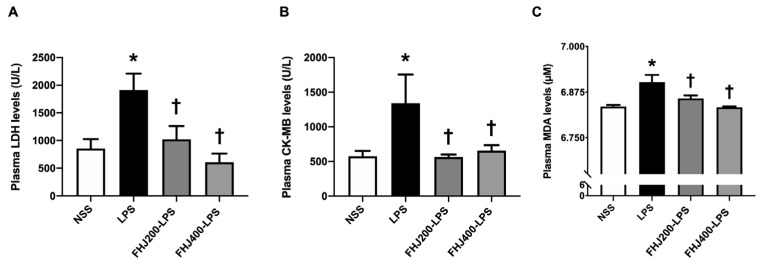
Effects of LPS injection and pretreatment with FHJ at 200 and 400 mg/kg for 2 weeks on cardiac injury markers and oxidative stress. (**A**) Plasma LDH levels, *n* = 8/group; (**B**) plasma CK-MB levels, *n* = 8/group; and (**C**) plasma MDA levels, *n* = 8/group. * *p* < 0.05 vs. NSS and ^†^ *p* < 0.05 vs. LPS (one-way ANOVA followed by the LSD post hoc test). Data are expressed as means ± S.E.M. NSS: rats treated with vehicle group; LPS: rats treated with a 12 h LPS injection; FHJ200-LPS: rats pretreated with 200 mg/kg of FHJ for 2 weeks followed by a 12 h LPS injection; and FHJ400-LPS: rats pretreated with 400 mg/kg of FHJ for 2 weeks followed by a 12 h LPS injection. LDH: lactate dehydrogenase; CK-MB: creatine kinase–myoglobin binding; MDA: malondialdehyde; FHJ: fermented *Houttuynia cordata* juice.

In rats treated with a 12 h LPS injection, cardiac injury was observed, as indicated by the LDH and CK-MK levels in plasma. Plasma LDH levels were significantly elevated in the LPS group in comparison with the NSS group (Figure 3A). In addition, there was a significant increase in plasma CK-MB in the LPS group in comparison with the NSS group (Figure 3B). Furthermore, plasma MDA levels were used to indicate oxidative stress, showing a significant increase in the LPS group in comparison with the NSS group (Figure 3C). This suggested that after 12 h, the injection of LPS led to the development of cardiac injury and elevated oxidative stress in rats.

Pretreatment with FHJ at doses of 200 and 400 mg/kg for 2 weeks reduced the levels of plasma LDH and CK-MB in LPS-injected rats back to the normal levels in comparison with the rats in the NSS group (Figure 3A,B). Regarding oxidative stress levels, administration of FHJ at 200 and 400 mg/kg also decreased the levels of plasma MDA in LPS-injected rats after 2 weeks of pretreatment in comparison with the LPS group (Figure 3C). Furthermore, the pretreatments with 200 and 400 mg/kg of FHJ shared the same efficacy in reducing the plasma MDA levels back to normal levels in comparison with the NSS group (Figure 3C). These data suggest that after 2 weeks, pretreatment with FHJ at 200 and 400 mg/kg demonstrated protective effects not only in reducing cardiac injury but also in decreasing oxidative stress in LPS-injected rats.

### 3.3. FHJ Reduced Cardiac Inflammation in Rats with LPS-Induced Sepsis

**Figure 4:**
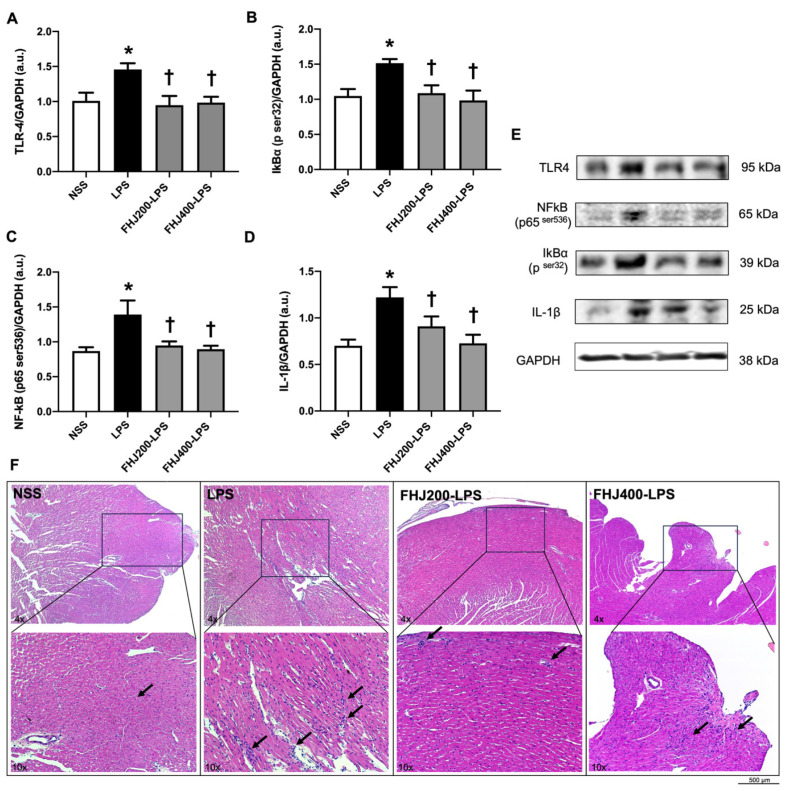
Effects of LPS injection and pretreatment with FHJ at 200 and 400 mg/kg for 2 weeks on cardiac inflammatory response. (**A**) Cardiac TLR-4 levels, *n* = 6/group; (**B**) cardiac p-IκB-α levels, *n* = 6/group; (**C**) cardiac p-NF-κB levels, *n* = 6/group; (**D**) cardiac IL-1β levels, *n* = 6/group; (**E**) representative Western blots; and (**F**) representative images of cardiac structural changes were captured by H&E staining and observed under 100x magnification. Black arrows indicate inflammatory cell in cardiac tissue. GAPDH was used as a loading control. * *p* < 0.05 vs. NSS and ^†^ *p* < 0.05 vs. LPS (one-way ANOVA followed by the LSD post hoc test). Data are expressed as means ± S.E.M. NSS: rats treated with vehicle; LPS: rats treated with a 12 h LPS injection; FHJ200-LPS: rats pretreated with 200 mg/kg of FHJ for 2 weeks followed by a 12 h LPS injection; and FHJ400-LPS: rats pretreated with 400 mg/kg of FHJ for 2 weeks followed by a 12 h LPS injection. TLR-4: toll-like receptor 4; NF-κB: nuclear factor kappa-light-chain-enhancer of activated B cells; IκB-α: inhibitor of nuclear factor of kappa-light-chain-enhancer in B-cell alpha; IL-1β: interleukin-1 beta; GAPDH: glyceraldehyde 3-phosphate dehydrogenase; H&E: hematoxylin and eosin; FHJ: fermented *Houttuynia cordata* juice.

Our results showed that the cardiac levels of TLR-4, a potent inflammatory receptor, were significantly increased in the LPS group compared with the NSS group (Figure 4A). Following this upregulation of the cardiac TLR-4 expression by LPS, the levels of inflammatory cytokines, including p-NF-κB p65, p-IκB-α, and IL-1β, were significantly increased in the hearts of the LPS group in comparison with those of the NSS group (Figure 4B–E). This suggested that the LPS injection successfully induced cardiac inflammation in rats.

Administration of FHJ at 200 mg/kg showed similar effects to those of the dose of 400 mg/kg after a 2-week pretreatment in reducing cardiac inflammatory receptors and markers in the LPS-injected rats. This was indicated by a decrease in the levels of TLR-4, p-NF-κB p65, p-IκB-α, and IL-1β in the cardiac tissue in LPS-injected rats, as they returned to the normal levels in comparison with the NSS group (Figure 4A–D). Representative Western blots are shown in Figure 4E. These results suggest that the pretreatments with FHJ at 200 and 400 mg/kg for 2 weeks exerted similar cardioprotective effects against LPS-induced cardiac inflammation in rats at the same levels. The myocardial sections were stained with H&E to assess cardiac damage. As depicted in Figure 4F, the NSS group exhibited no differences in cardiac morphology; in contrast, the LPS group displayed myocardial fiber fracture, cellular edema, and inflammatory cell infiltration. As expected, administration of 200 and 400 mg/kg of FHJ after a 2-week pretreatment significantly attenuated myocardial tissue damage in comparison with that in the LPS group.

### 3.4. FHJ Reduced Cardiac Apoptosis in Rats with LPS-Induced Sepsis

**Figure 5:**
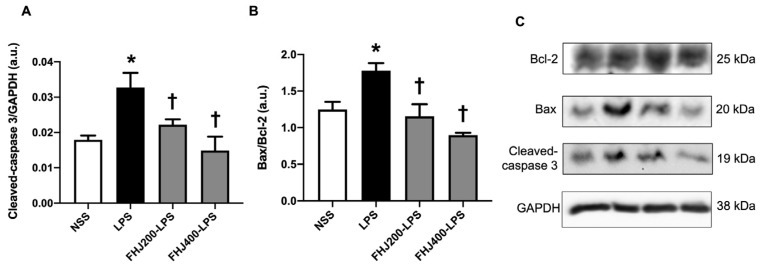
Effects of LPS injection and pretreatment with FHJ at 200 and 400 mg/kg for 2 weeks on cardiac apoptosis. (**A**) Cardiac cleaved-caspase 3/GAPDH, *n* = 6/group; (**B**) cardiac Bax/Bcl-2 levels, *n* = 6/group; (**C**) representative Western blots. GAPDH was used as a loading control. * *p* < 0.05 vs. NSS and ^†^ *p* < 0.05 vs. LPS (one-way ANOVA followed by the LSD post hoc test). Data are expressed as means ± S.E.M. NSS: rats treated with vehicle; LPS: rats treated with a 12 h LPS injection; FHJ200-LPS: rats pretreated with 200 mg/kg of FHJ for 2 weeks followed by a 12 h LPS injection; and FHJ400-LPS: rats pretreated with 400 mg/kg of FHJ for 2 weeks followed by a 12 h LPS injection. GAPDH: glyceraldehyde 3-phosphate dehydrogenase; Bcl-2: B-cell lymphoma 2; Bax: Bcl-2-associated X; FHJ: fermented *Houttuynia cordata* juice.

In addition to cardiac inflammation, we examined the levels of apoptotic cell death markers in the cardiac tissues of LPS-injected rats. This study demonstrated that the levels of cleaved-caspase 3/GAPDH were elevated in the LPS group (Figure 5A). Additionally, the cardiac Bax/Bcl-2 levels were increased in the LPS group (Figure 5B). This suggested that a 12 h LPS injection induced cardiac apoptosis in rats. However, pretreatment with FHJ at 200 and 400 mg/kg for 2 weeks alleviated both the cleaved-caspase 3/GAPDH and Bax/Bcl-2 levels in LPS-injected rats in comparison with the LPS group (Figure 5A,B). The representative Western blot bands depicting cardiac apoptosis are shown in Figure 5C. These data suggest that the administration of FHJ at 200 and 400 mg/kg exerted beneficial effects by protecting against apoptotic cell death in LPS-injected rats.

### 3.5. Summary of the Results

Table 2 summarizes the cardioprotective effects of the FHJ pretreatments at 200 mg/kg and 400 mg/kg on LPS-induced sepsis in rats. LPS significantly impaired cardiac function and increased injury markers (LDH and CK-MB), oxidative stress (MDA), inflammation (IL-1β and TLR-4/NF-κB), and apoptosis (cleaved caspase-3 and Bax/Bcl-2).

Pretreatment with FHJ prevented LPS-induced cardiac dysfunction and reduced these pathologic markers, with effects being observed at a minimum of 200 mg/kg in the rats. These results suggest that FHJ protects against LPS-induced cardiac dysfunction by attenuating inflammation, oxidative stress, and apoptosis. The individual *p*-values for these results are shown in Table 2.

## 4. Discussion

The main findings of this study are as follows: (1) A 12 h LPS injection induced sepsis and contributed to cardiac dysfunction in rats, as evidenced by reduced %LVEF and %LVFS values. These impaired cardiac functional parameters were associated with cardiac injury, inflammation, oxidative stress, and apoptosis. (2) Pretreatment with FHJ at doses of 200 and 400 mg/kg exerted a cardioprotective effect in rats with LPS-induced sepsis by suppressing cardiac inflammation and oxidative stress and improving cardiac function. (3) Pretreatment with FHJ at a dosage of 200 mg/kg was sufficient to show a beneficial effect in preventing and attenuating LPS-induced cardiac pathology in rats.

LPS is one of the most commonly used mediators for inducing models of sepsis [11,46,48,49,50,51]. It has been reported that LPS directly stimulates the activation of TLR-4, triggering an inflammatory response via the NF-κB signaling pathway [18,52,53]. The initiation of NF-κB activation is controlled by the phosphorylation of IκB-α, leading to its degradation and, consequently, upregulation of inflammatory cytokines, including TNF-α, IL-1β, and IL-6, which are considered hallmarks of sepsis [20,54,55,56]. Similarly, LPS stimulation has been shown to induce oxidative stress in sepsis models [57,58,59,60]. Evidence supporting the results of this study includes increased cardiac inflammatory markers, such as TLR-4, p-NF-κB, p-IκB-α, and IL-1β, along with elevated oxidative stress markers, as indicated by the MDA levels in LPS-injected rats. These changes are associated with cardiac apoptosis in LPS-induced sepsis models [61], as evidenced by the overexpression of cardiac cleaved-caspase 3 and Bax/Bcl-2 observed in this study. Furthermore, during sepsis, declines in heart contractility and myocardial damage have been observed [49,51]. Consistent with these findings, decreased levels of %LVEF and %LVFS, along with biomarkers of cardiac damage, were found in LPS-injected rats in this study. Thus, the administration of LPS successfully induced sepsis, leading to deleterious effects on cardiac function in rats.

There is evidence that products from HC have been widely used as dietary supplements in Southeast Asia, especially in Thailand, due to their numerous pharmacological properties, which include anti-inflammatory, anticancer, and antioxidant effects [34,35,36,62]. This study is the first to demonstrate the beneficial effects of pretreatment with FHJ on the heart in LPS-injected rats. Interestingly, pretreatment with FHJ at doses of 200 and 400 mg/kg for 2 weeks in LPS-induced cardiac inflammation in rats protected against inflammatory responses by reducing the expression of cardiac inflammatory proteins, including those involved in TLR-4 and NF-κB signaling, which led to a decrease in IL-1β. The FHJ was obtained from the same source as that used in the study by Sakuludomkan et al., who reported that their fermented HC product contained a variety of bioactive compounds, including gallic acid, chlorogenic acid, rosmarinic acid, rutin, quercetin, and beta-glucan [34]. Taken together, studies have demonstrated that products from HC are enriched with fatty acids, alkaloids, sterols, flavonoids, and phenolic acids [63,64]. Several studies have reported that flavonoids and phenolic acids exhibit potential anti-inflammatory and antioxidant effects [34,35,36,65]. These effects have been observed in inflammation-related pathological conditions both in vitro and in vivo [34,35,36,65]. Furthermore, evidence suggests that polyphenols, particularly those with a high percentage of phenolic compounds, can directly kill bacteria, such as Gram-negative bacteria that produce LPS, due to their hydrophobicity [66,67]. Another plausible effect of FHJ is that the fermentation process produces probiotics, which exert health-promoting functions by improving the nutraceutical value and enhancing the desirable compounds in herbal products [35,68,69]. A previous study also showed that fermented HC product increased the content of flavonoids, which have an excellent anti-inflammatory effect on LPS-treated macrophage cells [36]. Furthermore, several studies have highlighted the positive effects of phenolic compounds in improving cardiovascular function without causing adverse effects [70,71]. Therefore, as previously discussed, pretreatment with FHJ at a dose of at least 200 mg/kg for 2 weeks effectively protected against LPS-induced cardiac inflammation due to its bioactive properties.

Apoptosis, the most extensively studied type of cell death in the heart, is considered a factor contributing to impaired cardiac performance in sepsis models [28,72]. However, polyphenolic compounds, including flavonoids and phenolic acids, have been shown to effectively attenuate cardiac apoptosis in various pathological conditions [73,74]. In particular, reductions in oxidative stress and inflammation have been identified as crucial mechanisms for attenuating apoptosis and promoting cardiac protection [73,74]. Given the bioactive properties of FHJ, this is the first study to show that pretreatment with FHJ at doses of 200 mg/kg and 400 mg/kg for 2 weeks attenuated cardiac apoptosis by reducing the expression of cleaved-caspase 3 and Bax/Bcl-2 proteins. This effect might be attributed to the ability of flavonoids and phenolic acids to reduce MDA formation while stimulating the activity of antioxidant enzymes, such as superoxide dismutase and glutathione peroxidase [75,76]. Along with the anti-inflammatory effect of FHJ by reducing TLR-4, NF-κB, and IL-1β protein expression, this further contributes to its protective role in LPS-induced apoptosis in rats. These effects further attenuated cardiac injury by lowering the LDH and CK-MB levels in LPS-injected rats. Therefore, based on the positive outcomes discussed, pretreatment with FHJ led to an improvement in cardiac function in LPS-induced sepsis, as indicated by the increased %LVEF and %LVFS levels in rats. This study provides novel evidence suggesting that FHJ, a herb commonly found in Thailand, can be developed in the future as a potential treatment for patients with sepsis-related cardiovascular disease. Although the present study focused on pretreatment, further studies in the future should investigate the potential therapeutic effects of FHJ when administered after the onset of sepsis. Since this study focused primarily on the direct effects of FHJ on cardiac function in LPS-induced sepsis, systemic hemodynamics and blood pressure were not determined. This represents a limitation of the study, as systemic abnormalities may have influenced the observed improvements in cardiac function. Future studies could investigate the possible systemic effects of FHJ to provide a more comprehensive understanding of its therapeutic potential.

## 5. Conclusions

This study is the first to show that pretreatment with FHJ at a dose of at least 200 mg/kg effectively reduces cardiac inflammation, oxidative stress, and apoptosis, thus contributing to the prevention of LPS-induced cardiac dysfunction in rats. These results emphasize the potential of FHJ as a promising therapeutic approach for the treatment of sepsis-induced heart disease. The bioactive properties of FHJ suggest that it could be developed into a viable treatment option for patients with sepsis-related cardiac complications and that it represents a novel strategy for mitigating the deleterious effects of sepsis on the heart in clinical settings.

## Figures and Tables

**Table 1:** List of antibodies used in this study.

Name of Antibody	Manufacturer	Host	Dilution
Phosphorylation-NF-κB p65 (ser536)	Cell signaling	Rabbit	1:1000
TLR-4	Abcam	Rabbit	1:1000
Bcl-2	Merck	Rabbit	1:1000
Bax	Merck	Rabbit	1:1000
Cleaved-caspase 3	Cell signaling	Rabbit	1:1000
Phosphorylation-IκB-α (ser32)	Abcam	Rabbit	1:1000
IL-1β	Merck	Rabbit	1:1000
GAPDH	Sigma-Aldrich	Mouse	1:5000

NF-κB: nuclear factor kappa-light-chain-enhancer of activated B cells; TLR-4: toll-like receptor 4; Bcl-2: B-cell lymphoma 2; Bax: Bcl-2-associated X; IκB-α: inhibitor of nuclear factor of kappa-light-chain-enhancer in B-cell alpha; IL-1β: interleukin-1 beta; GAPDH: glyceraldehyde 3-phosphate dehydrogenase.

**Table 2:** A summary of the results of this study.

Parameters	Major Findings						
	NSS	LPS (vs. NSS)	* *p*-Value	FHJ200-LPS (vs. LPS)	† *p*-Value	FHJ400-LPS (vs. LPS)	† *p*-Value
%LVEF		↓	<0.0001	↑	<0.0001	↑	<0.0001
%LVFS		↓	<0.0001	↑	<0.0001	↑	0.0001
Plasma LDH		↑	0.0029	↓	0.0129	↓	0.0008
Plasma CK-MB		↑	0.0226	↓	0.0212	↓	0.0386
Plasma MDA		↑	0.0003	↓	0.0088	↓	0.0002
Cardiac TLR-4		↑	0.0106	↓	0.0067	↓	0.0105
Cardiac p-IκB-α		↑	0.0056	↓	0.0101	↓	0.0021
Cardiac p-NF-κB		↑	0.0013	↓	0.0057	↓	0.0026
Cardiac IL-1β		↑	0.0013	↓	0.0438	↓	0.0014
Cardiac cleaved-caspase 3		↑	0.0029	↓	0.0129	↓	0.0008
Cardiac Bax/Bcl-2		↑	0.0036	↓	0.0194	↓	0.0009

NSS: rats treated with vehicle; LPS: rats treated with a 12 h LPS injection; FHJ200-LPS: rats pretreated with 200 mg/kg of FHJ for 2 weeks followed by a 12 h LPS injection; FHJ400-LPS: rats pretreated with 400 mg/kg of FHJ for 2 weeks followed by a 12 h LPS injection; FHJ: fermented *Houttuynia cordata* juice; LVEF: left ventricular ejection fraction; LVFS: left ventricular fractional shortening; LDH: lactate dehydrogenase; CK-MB: creatine kinase–myoglobin binding; MDA: malondialdehyde; TLR-4: toll-like receptor 4; NF-κB: nuclear factor kappa-light-chain-enhancer of activated B cells; IκB-α: inhibitor of nuclear factor of kappa-light-chain-enhancer in B-cell alpha; IL-1β: interleukin-1 beta; Bcl-2: B-cell lymphoma 2; Bax: Bcl-2-associated X. ↑: an increase; ↓: a decrease. * *p* < 0.05 vs. NSS, and ^†^ *p* < 0.05 vs. LPS (one-way ANOVA followed by the LSD post hoc test).

## Data Availability

The original contributions presented in this study are included in the article. Further inquiries can be directed to the corresponding author(s).

## Abbreviations

The following abbreviations are used in this manuscript:

NSS	Normal saline solution
LPS	Lipopolysaccharide
FHJ	Fermented *Houttuynia cordata* juice
p.o.	Per oral
i.p.	Intraperitoneal injection
LVEF	Left ventricular ejection fraction
LVFS	Left ventricular fractional shortening
LDH	Lactate dehydrogenase
CK-MB	Creatine kinase–myoglobin binding
MDA	Malondialdehyde
TLR-4	Toll-like receptor 4
NF-κB	Nuclear factor kappa-light-chain-enhancer of activated B cells
IκB-α	Inhibitor of nuclear factor of kappa-light-chain-enhancer in B-cell alpha
IL-1β	Interleukin-1 beta
Bcl-2Bax	B-cell lymphoma 2Bcl-2-associated X
GAPDH	Glyceraldehyde 3-phosphate dehydrogenase
H&E	Hematoxylin and eosin
